# Length of hospital stay in involuntary admissions in Greece: a 10-year retrospective observational study

**DOI:** 10.1007/s00127-024-02653-x

**Published:** 2024-04-29

**Authors:** Maria Bakola, Vaios Peritogiannis, Konstantina Soultana Kitsou, Philippos Gourzis, Thomas Hyphantis, Eleni Jelastopulu

**Affiliations:** 1 Department of Public Health, Medical School, University of Patras, Rio, 26500 Patras, Greece; 2 Mobile Mental Health Unit of the Prefectures of Ioannina and Thesprotia, Society for the Promotion of Mental Health in Epirus, Ioannina, Greece; 3 Department of Psychiatry, Medical School, University of Patras, Patras, Greece; 4 Department of Psychiatry, Division of Medicine, School of Health Sciences, University of Ioannina, Ioannina, Greece

**Keywords:** Involuntary admissions, Compulsory admissions, Psychiatric patients, Mental disorders, Schizophrenia-spectrum disorders

## Abstract

**Purpose:**

The treatment of mental disorders has shifted from inpatient wards to community-based settings in recent years, but some patients may still have to be admitted to inpatient wards, sometimes involuntarily. It is important to maintain the length of hospital stay (LoS) as short as possible while still providing adequate care. The present study aimed to explore the factors associated with the LoS in involuntarily admitted psychiatric patients.

**Methods:**

A ten-year retrospective chart review of 332 patients admitted involuntarily to the inpatient psychiatric ward of the General University Hospital of Ioannina, Northwestern Greece, between 2008 and 2017 was conducted.

**Results:**

The mean LoS was 23.8 (SD = 33.7) days and was relatively stable over the years. Longer-stay hospitalization was associated with schizophrenia-spectrum disorder diagnosis, previous hospitalizations and the use of mechanical restraint, whereas patients in residential care experienced significantly longer LoS (52.6 days) than those living with a caregiver (23.5 days) or alone (19.4 days). Older age at disease onset was associated with shorter LoS, whereas no statistically significant differences were observed with regard to gender.

**Conclusion:**

While some of our findings were in line with recent findings from other countries, others could not be replicated. It seems that multiple factors influence LoS and the identification of these factors could help clinicians and policy makers to design more targeted and cost-effective interventions. The optimization of LoS in involuntary admissions could improve patients’ outcomes and lead to more efficient use of resources.

## Introduction

Contemporary mental health practice and policy implies that mental disorders should be treated in the less restricted environment, and treatment should be patient-centered, with a focus on rehabilitation [1]. Accordingly, over the last decades there was a shift in the treatment of mental disorders from inpatient wards and psychiatric hospitals to community settings. However, a comprehensive mental healthcare system should include both community- and hospital-based components of care, as suggested by the balanced care model [2]. Indeed, inpatient treatment may be required occasionally and sometimes involuntarily for the management of relapses and symptoms’ exacerbations, or for the containment of patients’ aggressive and violent behavior. When admission is unavoidable, length of hospital stay (LoS) should be kept as minimum as possible, but not too short, in order to maintain optimal hospital care. Indeed, a previous review of studies in high-income countries suggested that shorter LoS was associated with increased hospital readmissions [3].

The sociodemographic and clinical determinants of LoS in psychiatric admissions have been extensively studied. Duration of inpatient stay has been found to be related to age, gender, ethnicity, diagnosis, patients’ co-morbidities, severity of symptoms and patients’ functioning, among others [4, 5]. According to several studies, one of the strongest predictors of longer length of hospital stay is involuntary status at admission [6, 7] which in some countries is associated with particularly prolonged hospitalization [8]. Involuntary inpatient admissions and other coercive measures in psychiatry are considered the last resort for the treatment of patients who are at risk of further deterioration of their mental health, or may pose danger to themselves or others if left untreated [9]. In such cases, length of hospital stay should be sufficient for the effective management of an acute exacerbation of a severe mental illness, yet not too long, because prolonged inpatient treatment has been associated with adverse effects on patients [10]. However, research regarding the correlates of LοS in involuntary admissions is scarce [11].

In Greece, in the context of the ongoing progress of the psychiatric reform, deinstitutionalization and development of community mental health services have advanced significantly over the last decades, whereas several psychiatric units in general hospitals have been launched across the country [12, 13]. However, the main target of reform has been the closure of asylums and public mental hospitals and the transfer of patients to community-based residential care [13] and despite the aforementioned progress, specialized outpatient services are still under-developed [12]. Notably, in countries with well-organized community mental healthcare, such as the United Kingdom, a rise in involuntary admissions has been previously reported, as a consequence of ongoing deinstitutionalization processes [14]. Furthermore, during the last decade’s economic crisis patient visits to emergency psychiatric departments, outpatient wards and mental health clinics in the national healthcare system had been significantly increased [12]. Subsequently, a trend toward the increase of involuntary admissions over the last years has been reported in some regions of Greece [15]. Accordingly, there is growing research on involuntary admissions recently [16–18], and research has shown that involuntary admission rates may differ significantly across the country. That is, rates vary considerably and are higher in metropolitan locations, such as the capital of Greece, Athens and the second largest city, Thessaloniki, than in smaller cities (ranging from 24.4% to 57.2%) [19]. Mean LoS in involuntary admissions also varies considerably, from 18.5 days in Thessaloniki, up to 41.8 days in Patras, Southwest Greece [19, 20]. With regard to the site of the present study, the inpatient ward of the University General Hospital of Ioannina (UGHI), we have recently reported on the rate of involuntary admissions over a decade, which is one of the lowest in Greece (24.3%), whereas the mean LoS has been 23.8 days [21].

Although recent research attempted to investigate the clinical profiles of patients that could be associated with admission status [22] to the best of our knowledge there are no data in Greece with regard to the potential factors that may be related to LoS in involuntary admissions. The objective of the present study was therefore to explore the associations of LoS in patients subjected to involuntary hospitalizations in the psychiatric ward of a large university teaching hospital, namely the UGHI, Northwestern Greece, with clinical and sociodemographic characteristics. Our hypothesis was that factors that are related to longer LοS in involuntarily admitted psychiatric patients, would be similar with those encountered in voluntary admissions.

## Methods

### Design and study sample

**Fig. 1:**
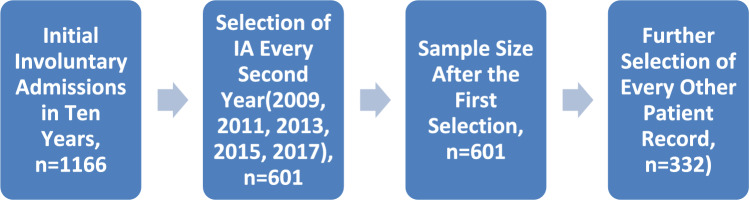
Selection of patient files for involuntary admissions to psychiatric ward

The details of data acquisition and processing have been previously reported [21]. In summary, in this retrospective study, all records of involuntary admissions (n = 1,166) in the psychiatric ward of the UGHI, Northwestern Greece, over a 10-year period (from January 2008 to December 2017) were considered. All patients’ medical records were handwritten, paper-based, and spread across different branches of the ward, which made it difficult to access all of them. To overcome this problem, we used a systematic sampling method to obtain a representative sample of patients during the study period. We further selected involuntary admissions of every second year, for the years 2009, 2011, 2013, 2015, 2017 (n = 601), and of those, every other patient record (n = 332) was retrieved for in-depth examination (Fig. 1). When confronted with missing data, we selected the subsequent available record from the list. Our aim was to capture at least 50% of patients who experienced involuntary admission during the specified years. Similar methodology for sample selection has been previously used in research [23, 24].

### Measurements

#### Dependent variable

The dependent variable in the study was the LoS, defined as the duration of involuntary hospitalization of patients in the psychiatric ward. The LoS for each admission was calculated as the number of days that elapsed between admission and discharge, on the basis on the difference between the dates of admission to and discharge from the ward [4, 6].

#### Independent variables

The independent variables included the individual characteristics of the patients. For each patient who was admitted involuntarily, we extracted socio-demographic and clinical data, such as age, gender, place of residence, marital status, living situation, occupational status, education, psychiatric diagnosis and illness duration. The age of each patient was expressed in years. Gender was categorized as male or female based on biological sex. Marital status was classified into three groups: single, married, or divorced. Education was divided into three categories: primary education (≤ 9 years), secondary education (10–12 years), and tertiary education (> 12 years). Occupational status was categorized as either employed or unemployed. Psychiatric diagnoses were made according to the criteria of the 10th revision of the World Health Organization's International Classification of Diseases (ICD-10) for the classification of mental health disorders. Those included organic brain syndromes; substance abuse disorders; schizophrenia-spectrum disorders; mood disorders; anxiety disorders; personality disorders; mental retardation and disorders of psychological development. In routine clinical practice of the ward diagnoses are made according to the usual clinical examination, taking into account all the available information from caregivers and referring primary care physicians. No structured interview is regularly used to determine the diagnosis. Illness duration was retrieved from patients’ charts, referring to the interval from official initial diagnosis to involuntary hospitalization. Due to the heterogeneity of the study sample in terms of diagnosis, other information such as comorbidities and medication was not searched for.

#### Ethical approval

Ethical approval for the present study was obtained from the Institutional Scientific Board of the University General Hospital of Ioannina.

#### Statistical analysis

Continuous variables were presented as mean ± standard deviation (SD), whereas categorical variables were presented as number (percentage). To examine differences in categorical variables, either the chi-square test or Fisher's exact test was used to determine whether the proportions between the values of one categorical variable were significantly different from those of another categorical variable. For comparisons between two independent groups, when the dependent variable was either ordinal or continuous but not normally distributed, the chi-square test and Mann–Whitney U test were used. Normality of variables was tested with the Shapiro-Wilks test. In all cases, statistical significance was set at p < 0.05. Statistical analyses were performed using SPSS version 25.0 (SPSS Inc., Chicago, IL, USA).

## Results

During the decade studied, a total of 1,166 involuntary admissions were recorded, representing 24.3% of the total admissions (4,810) in the UGHI psychiatric ward. By applying systematic sampling as aforementioned, a total of 332 patients who were involuntarily admitted were further processed for analysis, representing 28.5% of the total involuntary admissions over the study period. The sample consisted mostly by male (more than two-thirds of patients), middle-aged patients (mean age 49 ± 13 years), with relatively late illness onset (illness duration 3.9 ± 7.5 years). The majority of the patients were single and lived with their parents or siblings, while 16.6% lived with their own family, and 3% were resided in residential care. Slightly more than 40% had up to 9 years of education and almost two-thirds were unemployed. More than half of the patients (n = 186, 56%) had been diagnosed with a schizophrenia-spectrum disorder. The mean age of diagnosis in those patients was 39.1 ± 13.3 years and the mean illness duration was 5 ± 8.4 years. Table 1 presents the sociodemographic and clinical characteristics of the patients.

**Table 1:** Patients’ sociodemographic and clinical characteristics (N = 332)

Length of hospital stay, in days, mean (± SD)	23.8 (± 33.7)	
Illness duration, in years, mean (± SD)	3.9 (± 7.5)	
Age, in years, mean (± SD)	49 (± 13)	
	n	%
Gender		
Male	224	67.5
Female	108	32.5
Place of residence		
Ioannina and affiliated prefectures	216	65.1
Rest of Greece	114	34.3
Other country	2	0.6
Nationality		
Greek	301	90.7
Other	31	9.3
Marital status		
Single	233	70.2
Married	73	22.0
Divorced	26	7.8
Living with		
Parents/siblings	202	60.8
Living with own family	55	16.6
Residential care	10	3.0
Other	3	0.9
Missing info	62	18.7
Educational status		
Primary education (≤ 9 years)	139	41.9
Secondary education (9–12 years)	121	36.4
Tertiary education	72	21.7
Occupational status		
Employed	117	35.2
Unemployed	215	64.8
Diagnosis coded with ICD-10*		
F00-F09	6	1.8
F10-F19	31	9.3
F20-F29	186	56.0
F30-F39	65	19.6
F40-F48	9	2.7
F60-F69	12	3.6
F70-F72	7	2.1
Other	16	4.8

^*^Explanation of Diagnosis ICD-10 codes:

**F00-F09:** organic, including symptomatic, mental disorders

**F10-F19:** mental and behavioural disorders due to psychoactive substance use

**F20-F29:** schizophrenia, schizotypal and delusional disorders

**F30-F39:** mood [affective] disorders

**F40-F48:** neurotic, stress-related and somatoform disorders

**F50-F59:** behavioural syndromes associated with physiological disturbances and physical factors

**F60-F69:** disorders of adult personality and behaviour

**F70-F79:** mental retardation

**F80-F89:** disorders of psychological development

**F90-F98:** behavioural and emotional disorders with onset usually occurring in childhood and adolescence

**F99-F99:** unspecified mental disorder

**Fig. 2:**
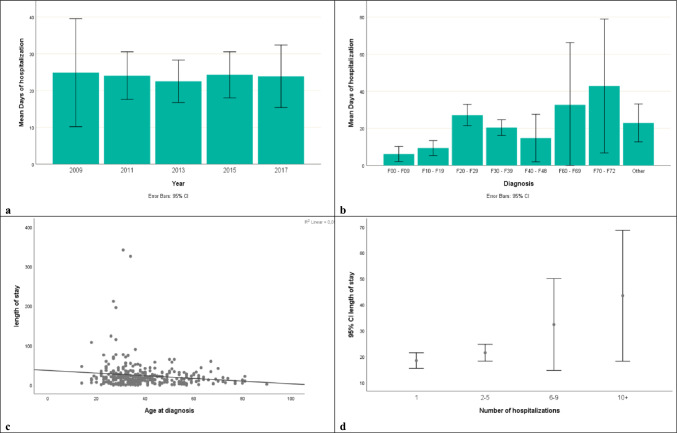
**a** Mean length of hospital stay in involuntary admissions by year; **b** Mean length of hospital stay in involuntary admissions by diagnosis (**F00-F09:** organic, including symptomatic, mental disorders, **F10-F19:** mental and behavioural disorders due to psychoactive substance use, **F20-F29:** schizophrenia, schizotypal and delusional disorders, **F30-F39:** mood [affective] disorders, **F40-F48:** neurotic, stress-related and somatoform disorders, **F50-F59:** behavioural syndromes associated with physiological disturbances and physical factors, **F60-F69:** disorders of adult personality and behavior, **F70-F79:** mental retardation, **F80-F89:** disorders of psychological development, **F90-F98:** behavioural and emotional disorders with onset usually occurring in childhood and adolescence, **F99-F99:** unspecified mental disorder); **c** The correlation of length of stay in involuntary admitted patients with age at disease onset; **d** The correlation of length of stay in involuntary admitted patients with the number of previous admissions

**Fig. 3:**
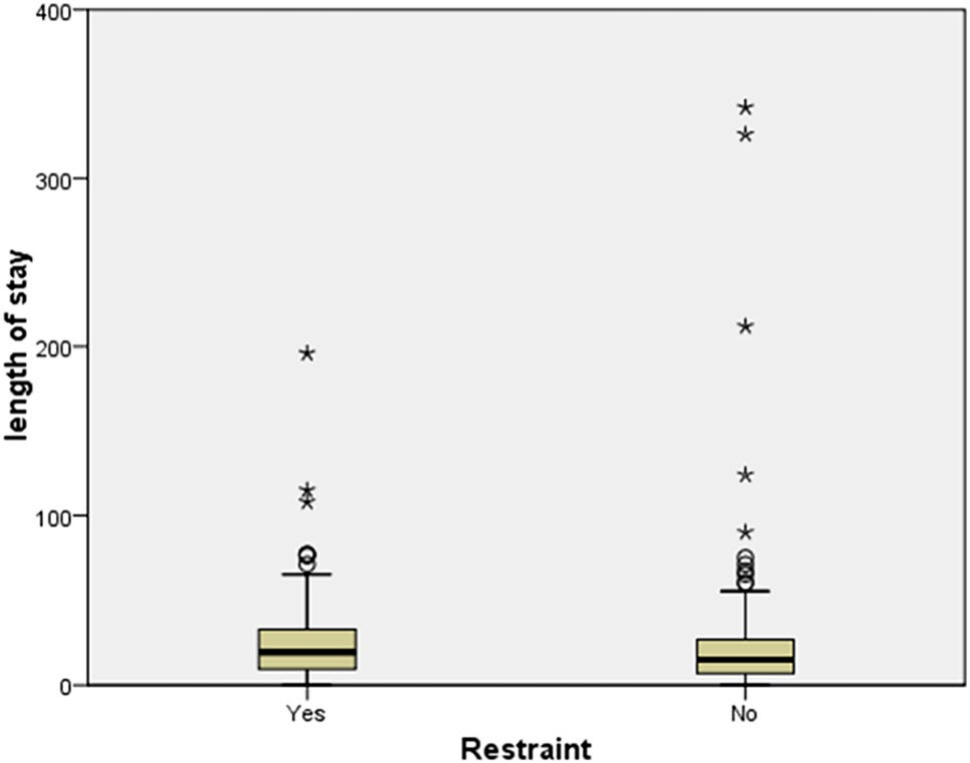
The correlation of length of hospital stays with the application of mechanical restraint in involuntary admitted patients

The duration of hospital stay over the years is depicted in Fig. 2a and its correlations are presented in Table 2. A Kruskal–Wallis test indicated that the days of hospitalization differed with regard to diagnosis (Fig. 2b), whereas LoS was also correlated with the age at onset of the disease (Fig. 2c) and the number of previous hospitalizations (Fig. 2d). The difference between males and females in terms of the duration of hospitalization for all patients, as well as for those admitted for the first time was not statistically significant. Similarly, there was no statistical significance of the average duration of hospitalization in relation to the patient’s age at the time of admission. In cases that mechanical restraint was applied, the duration of hospitalization was statistically significantly higher, compared to the cases of patients who were not subjected to mechanical restraint (26.8 vs 22.4 days) (Fig. 3). Finally, a Kruskal–Wallis test indicated that patients in residential care had a significant longer LoS than patients living with a caregiver or patients living alone (52.6 days vs 23.5 vs 19.4, respectively).

**Table 2:** Co-relations of length of hospital stay in involuntarily admitted patients

	Length of hospital stay	
	Statistical test	P
Diagnosis	H(7) = 35.734	< 0.001
Age of disease onset	rs(332) = − .113	0.040
Number of hospitalizations	rs(332) = .140	0.010
Gender		
All patients	Z = − 0.289	0.772
First-admitted patients	Z = − .721	0.471
Mechanical restraint	Z = − 2.529	0.011
Age at admission	rs(332) = − .037	0.504
Type of care	H(2) = 12.638	0.002

## Discussion

The present study aimed to address the duration of hospitalization in patients subjected to involuntary admissions and to explore the correlations of LoS with clinical and demographic variables. It was found that LoS had been relatively stable over the 10-year study period and it was correlated with diagnosis; age at disease onset; the number of previous hospitalizations; mechanical restraint; and accommodation to residential care. It appeared that the diagnoses of schizophrenia-spectrum disorders, personality disorders and intellectual disability were associated with longer hospitalizations in the present sample of patients, while the duration of hospitalization was found to be inversely correlated to the age of patients at the time of diagnosis, that is the older the patient when diagnosis was made, the shorter the LoS. Patients with more previous admissions had significantly longer hospitalizations, as well as those residing in residential facilities. In cases that further coercive measures were applied, that is mechanical restraint, the duration of hospitalization was statistically significantly higher, compared to patients who were not subjected to mechanical restraint. No correlations of LoS with gender and age at admission were found in this study.

In line with previous research in various countries [25–27], schizophrenia-spectrum disorders have been correlated to increased hospital stay in the present study. Additionally, a significant association of LoS with intellectual disabilities and personality disorders was found. In the case of the former, the patients that were encountered were only 7 and conclusions cannot be reached. Similar small number of patients (n = 12) with personality disorders were included in the analysis, making the interpretation of findings difficult. However, the finding that hospital stay in patients with personality disorders is prolonged may be clinically relevant, because those patients may be particularly disrupting for psychiatric wards [28]. Previous research has shown that the diagnosis of personality disorder is associated with shorter LoS in voluntary hospitalizations [4]. This trend may differ in involuntary admissions, but further research is needed to clarify this finding.

Duration of hospitalization was found to be inversely correlated to the age of patients at the time of diagnosis. This finding is probably explained by the prevalence of psychiatric diagnoses across the lifespan. Several severe mental disorders that are associated with prolonged hospitalizations, such as schizophrenia-spectrum disorders are mostly begin and diagnosed at younger age [29, 30].  It should be noted however, that in the present study the mean age of patients with schizophrenia-spectrum disorders at the time of diagnosis had been older (39.1 ± 13.3 years), compared with data from the previous epidemiological study of first episode of psychosis in our catchment area [31]. Although this finding could be attributed to methodological issues, that are missing information with regard to the disorders’ onset, further study is warranted to interpret this finding. On the other hand, older patients with age-related disorders, such as dementia, and other syndromes, such as late-onset delusional disorder may be involuntarily admitted for the management of agitation and psychotic symptoms. In those cases, however, it is possible that patients can be discharged earlier than younger patients with schizophrenia-spectrum disorders, who may pose a greater perceived threat for their families or others. Current patients’ age was not correlated with LoS in this study, in line with recent research [32]. However, evidence with regard to the association of age with LoS is conflicting, as other studies suggest that older age is associated with increased LoS [33, 34].

A history of previous admissions was found to be associated with longer hospitalization in the present study. This finding has been consistently reported in the literature [4, 25], and may be partly explained by taking into account the diagnoses of multi-admitted patients. Such patients are more likely to suffer a severe mental disorder, such as schizophrenia or bipolar disorder, that have been associated with longer hospital stays, as aforementioned. It should be also noted that the very long-term outcome of psychotic disorders is often poor for a large proportion of patients [35, 36] and this could be reflected to repeated and longer involuntary hospitalizations over time. Furthermore, over the course of a severe mental illness patients are at increased risk of developing physical morbidity [37, 38], which has been associated with psychiatric readmissions [39]. In such cases, hospitalization may be prolonged due to laboratory examinations and co-operation with other medical specialties for the optimal management of physical morbidity. Indeed, it has been previously reported that medical co-morbidity is at least weakly associated with increased length of hospitalization in psychiatric patients [40]. It should be noted, however, that data on patients’ physical morbidity were not retrieved in the present study.

Further application of coercive measures, such as restraint, was associated with longer LoS in the present study. This finding is in line with previous research [34], and may be relevant in involuntary admissions, because they have been consistently associated with the application of restraint and seclusion [41, 42]. Patients living in residential facilities had a significant longer LoS than patients living with a caregiver or patients living alone, suggesting that when these patients need to be involuntarily hospitalized, their hospitalization may be prolonged, a finding that has been consistently replicated in the literature [26, 27, 34]. Some potential confounders associated with the observed outcomes should be considered. That is, the application of restraint, which was associated with longer LoS, could be the result of a more severe illness. Indeed, there is evidence that longer hospital stays may be associated with higher illness severity [4, 43, 44]. However, illness severity could not be determined in the present retrospective study. Longer LoS in those receiving residential could be also associated with discharge queueing at the care homes and the discharge planning that should be made. For patients that already live in residential facilities early return to the facility may not be feasible, because if they still have symptoms, they could be disruptive for the facility environment. Furthermore, relapse in those patients supposedly occurred despite receiving treatment as prescribed, meaning that medication switching may be warranted during their hospitalization. Accordingly, longer hospital stay may be required for patients’ monitoring under the new treatment regimen. Patients with caregivers seem to be more easily discharged in the present study. This finding differs from other research which suggests that, in the case of patients with severe mental disorders, having a caregiver may be associated with longer hospital stay, in order to preserve caregivers’ health and wellbeing [4].

## Limitations

This study has some limitations. Despite the adequate number of cases examined, some diagnoses and other variables involved only a small number of patients, thus making statistical analysis inapplicable or inconclusive. Furthermore, due to the retrospective design of the study some information may have been missed. Finally, other factors that may have influenced the result, such as co-morbidities and medication use were not inquired for.

Although LoS is viewed as an indicator of quality of mental healthcare, determining the ideal LoS for psychiatric patients is still a challenge. Inappropriate reductions in LoS can impact on quality of care and affect the outcomes of patients, and may increase readmission rates. Differences in LoS across settings may reflect differences in patient needs, but may also be indicative of differences in practice patterns and in efficiency of care provision [4]. Moreover, the definition of short or long LoS in voluntary and involuntary admissions varies considerably across studies in different settings and countries and remains a limitation in the literature. Several studies have used a binary variable to define short or long LoS. For instance, in a study in Malawi LoS was considered long when exceeded 28 days [45], whereas in a Brazilian study the limit was set at 20 days [8]. In the present study, as well as in other research LoS was examined as a continuous variable.

## Implications for care

The study of the correlates of length of inpatient stay in involuntary hospitalizations is important and could guide the interventions to reduce the duration of hospital stay. It is particularly relevant to elucidate modifiable factors that affect LoS in involuntary admissions, such as those related to the health system. Previous research has shown that strong positive correlations with LoS were inpatient capacity and human resources of healthcare professionals [46]. This may mean that optimizing bed availability and staffing in inpatient wards could help optimizing LoS as well. Legal procedures may also affect LoS in involuntary admissions, which may be prolonged due to the required court proceedings to continue hospitalization [47]. Conceivably, simplifying and hastening legal procedures could reduce LoS, without compromising patients’ care. Moreover, longer-stay hospitalization has been linked with lack of discharge planning [27], meaning that discharged patients may not adequately referred to outpatient services. Subsequently, a comprehensive discharge planning that would enable continuity of care across inpatient and community mental healthcare settings could result in reduction of LoS. Yet, there is some evidence that continuity of care in patients with schizophrenia, that are mostly involved in involuntary admissions and may have the longest hospitalizations, declines significantly over the years, and that has been associated with adverse outcomes [48].

Interventions to reduce LoS should target to the operational level and the organization of effective community mental health services. Current practice implies that when a psychiatric patient requires hospitalization, it should be as brief and as efficient as possible. It has been argued that, in order to achieve this goal, the community and the healthcare system should provide resources such as psychosocial care centers and also promote the integration of mental health into primary healthcare [8]. In our region, the locally-based Mobile Mental Health Unit of the prefectures of Ioannina and Thesprotia in rural areas has been shown to significantly reduce the number of involuntary and voluntary admissions, and the duration of hospital stay in both types of admission in patients with psychotic disorders [49]. These findings were recently replicated in insular Greece [50], and are in line with previous research in Athens, that suggested that the launch of a community mental health service had been associated with a significant reduction in the number and days of hospitalization, as well as a reduction in involuntary admissions [51]. Other research has addressed the impact of Assertive Community Treatment (ACT) on admissions and LoS in patients with severe mental illness. A previous meta-analysis suggested that patients receiving ACT were less likely to be admitted to hospital than those receiving standard community care and spent less time in hospital [52]. A more recent study in Denmark found that ACT decreased the number of voluntary and involuntary hospitalizations, and that inpatient stay was reduced to more than half over a 2-year period [53].

The reduction of LoS is relevant from an economic perspective but also from patients’ perspective, as prolonged hospital stay not only increases the costs of care, but also disrupts the patient’s life and alienates patients from their environment [11]. A better understanding of factors associated with LoS might help identifying patients at higher risk of longer hospital stay, thus allowing mental health professionals to allocate these patients to more targeted and cost-effective services. Accordingly, secondary effects of long-term hospitalization and unnecessary costs could be avoided [6].

Elucidating the factors that are associated with LoS in involuntary admissions is important, if we are to better understand the mechanisms of those hospitalizations and improve inpatient care. The present study is one of the few on the topic, concerning general hospital psychiatric wards and adds to a limited literature. Further research on the duration of involuntary admissions is warranted; the factors that determine LoS; and the most appropriate interventions to reduce LoS in involuntary hospitalizations, without compromising quality of inpatient care.

## Conclusion

The present study suggests that younger age, previous hospitalizations, use of mechanical restraint and living in residential care are associated with longer length of hospital stay in involuntary admitted patients. While some of our findings were in line with recent findings from other countries, others could not be replicated. It seems that multiple factors influence LoS and the identification of these factors could help clinicians and policy makers to design more targeted and cost-effective interventions. The optimization of LoS in involuntary admissions could improve patients’ outcomes and lead to more efficient use of resources. However, due to the scarcity of data on LoS in involuntary admissions and the differences across different settings and countries, research should be continued worldwide.

## Data Availability

The data is confidential and may be available upon reasonable request.
